# Hypoxia-Induced *MALAT1* Promotes the Proliferation and Migration of Breast Cancer Cells by Sponging *MiR-3064-5p*

**DOI:** 10.3389/fonc.2021.658151

**Published:** 2021-05-03

**Authors:** Chung-Hsien Shih, Li-Ling Chuang, Mong-Hsun Tsai, Li-Han Chen, Eric Y. Chuang, Tzu-Pin Lu, Liang-Chuan Lai

**Affiliations:** ^1^ Graduate Institute of Physiology, College of Medicine, National Taiwan University, Taipei, Taiwan; ^2^ School of Physical Therapy and Graduate Institute of Rehabilitation Science, College of Medicine, Chang Gung University, Taoyuan, Taiwan; ^3^ Department of Physical Medicine and Rehabilitation, Chang Gung Memorial Hospital, Taoyuan, Taiwan; ^4^ Institute of Biotechnology, National Taiwan University, Taipei, Taiwan; ^5^ Bioinformatics and Biostatistics Core, Center of Genomic and Precision Medicine, National Taiwan University, Taipei, Taiwan; ^6^ Institute of Fisheries Science, College of Life Science, National Taiwan University, Taipei, Taiwan; ^7^ Graduate Institute of Biomedical Electronics and Bioinformatics, National Taiwan University, Taipei, Taiwan; ^8^ Collage of Biomedical Engineering, China Medical University, Taichung, Taiwan; ^9^ Institute of Epidemiology and Preventive Medicine, National Taiwan University, Taipei, Taiwan

**Keywords:** hypoxia, long non-coding RNA, *MALAT1*, breast cancer, hypoxia inducible factor-1α, microRNA, *miR-3064-5p*

## Abstract

Hypoxia, a common process during tumor growth, can lead to tumor aggressiveness and is tightly associated with poor prognosis. Long noncoding RNAs (lncRNAs) are long ribonucleotides (>200 bases) with limited ability to translate proteins, and are known to affect many aspects of cellular function. One of their regulatory mechanisms is to function as a sponge for microRNA (miRNA) to modulate its biological functions. Previously, *MALAT1* was identified as a hypoxia-induced lncRNA. However, the regulatory mechanism and functions of *MALAT1* in breast cancer are still unclear. Therefore, we explored whether *MALAT1* can regulate the functions of breast cancer cells through miRNAs. Our results showed the expression levels of *MALAT1* were significantly up-regulated under hypoxia and regulated by HIF-1α and HIF-2α. Next, in contrast to previous reports, nuclear and cytoplasmic fractionation assays and fluorescence *in situ* hybridization indicated that *MALAT1* was mainly located in the cytoplasm. Therefore, the labeling of *MALAT1* as a nuclear marker should be done with the caveat. Furthermore, expression levels of miRNAs and RNA immunoprecipitation using antibody against AGO2 showed that *MALAT1* functioned as a sponge of miRNA *miR-3064-5p*. Lastly, functional assays revealed that *MALAT1* could promote cellular migration and proliferation of breast cancer cells. Our findings provide evidence that hypoxia-responsive long non-coding *MALAT1* could be transcriptionally activated by HIF-1α and HIF-2α, act as a miRNA sponge of *miR-3064-5p*, and promote tumor growth and migration in breast cancer cells. These data suggest that *MALAT1* may be a candidate for therapeutic targeting of breast cancer progression.

## Introduction

Several studies have confirmed that the tumor microenvironment promotes cancer progression in many ways, especially *via* therapeutic resistance. Hypoxia is a common feature of malignant tumors (1). It has been described as a complicated incident of the tumor microenvironment that promotes tumor aggressiveness and metastasis (2, 3), and is strongly associated with poor prognosis (4, 5). Hypoxia is harmful to cancer cells, but it drives their adaptation, thereby promoting malignant progression (6, 7). In response to hypoxia, cancer cells exhibit modified expression of numerous genes regulated by hypoxia-inducible factors (HIFs), the major components of hypoxia signaling pathways (8). Most of the HIF-dependent responses rely on changes in the expression of genes associated with angiogenesis, proliferation, epithelial to mesenchymal transition, and metastasis (9). These changes allow malignant cells to survive the harsh hypoxic environment. However, the details of how hypoxia leads to tumor progression remain to be identified.

Long noncoding RNAs (lncRNAs) are transcripts which are longer than 200 nucleotides but have limited protein-coding capacity (10). Emerging evidence has shown that lncRNAs are a critical factor for both normal development and tumorigenesis (11, 12), and that they participate in epigenetic regulation of gene expression (13, 14). In recent studies, lncRNAs such as metastasis-associated lung adenocarcinoma transcript 1 (*MALAT1*) have been shown to participate in cancer progression. *MALAT1* was initially identified as being up-regulated in primary human non-small cell lung cancer cells with higher metastasis ability (15), and was associated with metastasis and survival of cancer cells (14). Later, it was observed to have aberrant expression levels in many cancers (16, 17), and to be involved in post−transcriptionally modified primary transcripts and regulated gene expression (18). Although hypoxia-inducible factor was a major regulator of the non-coding and coding transcriptome in hypoxia (19), the regulatory mechanism of *MALAT1* in breast cancer cells remains to be clarified. In addition to lncRNA, microRNAs (miRNAs), a class of small non-coding RNA transcripts (~22 nucleotides), also regulate the gene expression levels by binding to the 3'-untranslated regions (3'-UTRs) of target mRNAs (20, 21). Many studies have reported that miRNAs are differentially expressed in hypoxia and related to various aspects of cancer pathogenesis and progression, such as cell differentiation, proliferation, migration, invasion, apoptosis, and drug resistance (22–28). Some studies have reported the interaction between lncRNA and miRNA, specifically that lncRNA can be competing endogenous RNA by acting as a sponge for miRNA (29, 30).

Previously, we used next-generation sequencing (NGS) technology to identify oxygen-responsive lncRNAs in MCF-7 breast cancer cells and identified *MALAT1* as one of the top five up-regulated lncRNAs under hypoxia. However, the regulatory mechanism and function of *MALAT1* in breast cancer are not known. Therefore, the purpose of this study was to explore the regulatory mechanisms and functions of *MALAT1* in breast cancer cells. Expression levels of *MALAT1* in MCF-7 under *HIF-1A* or *HIF-2A* overexpression were examined by quantitative RT-PCR (qPCR). Endogenous expression levels of *MALAT1* in MCF-7 cells grown at different oxygen concentrations were examined by qPCR. Luciferase reporter assays verified the direct interaction between HIF-1α or HIF-2α and the putative hypoxia response elements in the *MALAT1* promoter. To confirm the distribution of *MALAT1* in breast cancer cells, nuclear-cytoplasmic RNA fractionation assays and RNA fluorescence *in situ* hybridization (FISH) were performed. To identify miRNAs affected by *MALAT1*, NGS was performed in *MALAT1*-knockdown cells under hypoxia, followed by RNA immunoprecipitation (RIP) assays using antibody against AGO2 protein, the essential component of the miRNA-induced silencing complex, and by qPCR. Furthermore, a series of functional assays of *MALAT1* were performed. The results indicate a role for *MALAT1* as a sponge for miRNA, which increases the metastatic potential of MCF-7 breast cancer cells.

## Materials and Methods

### Cell Culture and Treatments

MCF-7 and MDA-MB-231 breast cancer cells and HEK293T human embryonic kidney cells were maintained in Dulbecco’s Modified Eagle Medium (DMEM) (GIBCO, Carlsbad, CA, USA). All were supplemented with 10% fetal bovine serum (HyClone, Logan, UT, USA) and 1% antibiotics (penicillin-streptomycin) (GIBCO). Human mammary epithelial cell line MCF-10A was maintained in Dulbecco’s Modified Eagle Medium: Nutrient Mixture F-12 (DMEM/F12) (GIBCO) containing horse serum (5%), epidermal growth factor (20 ng/ml), hydrocortisone (0.5 mg/ml) (Sigma, Saint Louis, MO, USA), cholera toxin (100 ng/ml) (Sigma), insulin (10 μg/ml) (Sigma) and 1% antibiotics (penicillin-streptomycin) (GIBCO). Cells were incubated at 37°C in a humidified incubator with 5% CO_2_. In some experiments, cells were treated with 300 μM cobalt (II) chloride (Sigma) to mimic hypoxic conditions or cultured in a hypoxia chamber (Ruskinn Technology, Bridgend, UK) filled with a gas mixture of 0.5% O_2_, 5% CO_2_ and 94.5% N_2_ for 24 h.

### Plasmid Constructs

To overexpress *MALAT1*, the expression plasmid pCMV-*MALAT1* was kindly provided by Dr. Yi-Ching Wang, National Cheng Kung University, Taiwan. To overexpress HIF-1α and HIF-2α under normoxic conditions, pcDNA3-HIF-1α-P402A/P564A (Addgene plasmid #18955) and pcDNA3-HIF-2α-P405A/P531A (Addgene plasmid #18956) were bought from Addgene (Cambridge, MA, USA). The mutations produce proteins that resist O_2_-regulated prolyl hydroxylation in the oxygen-dependent degradation domain and are thus stable under normoxia. To determine promoter activity by luciferase assay, the luciferase expression plasmid pGL3 was purchased from the Biomedical Resource Core of the 1^st^ Core Facility Lab, National Taiwan University (NTU) College of Medicine (Taipei, Taiwan). Briefly, the *NDRG1-OT1* promoter region encompassing -1 ~ -2,000 bp relative to the transcription start site of *MALAT1* was amplified from human genomic DNA by PCR and subcloned into the pGL3-basic vector to create the pGL3-*MALAT1* promoter plasmid.

To determine the binding activity of *miR-3064-5p* on *MALAT1*, luciferase expression plasmids with mutations at the binding sites pmiR-GLO-*MALAT1* S1 (1,279 - 1,302 bp) and pmiR-GLO-*MALAT1* S2 (7,837 - 7,860 bp) were purchased from the Biomedical Resource Core of the 1^st^ Core Facility Lab (NTU).

### Lentiviral shRNAs

Lentiviral plasmids pLKO_TRC005_sh*MALAT1* #1 and pLKO_TRC005_sh*MALAT1* #2 encoded short hairpin RNA (shRNA) against *MALAT1* (GeneID: 378938). The oligonucleotides synthesized for these shRNAs were as follows: sh*MALAT1* #1: 5'- GAG CGA AAG GAT GCC CAT CCG CCC TTT TTG AAT TCT AGA TCT TGA GAC AAA-3' (sense), 5'-GAG CGA AAG GAT GCC CAT CCG CCC CCG GTA CCT CGT CC-3' (antisense); shMALAT1 #2: 5'-GAG AGA GGG AAG CTC GTT AGT GCC TTT TTG AAT TCT AGA TCT TGA GAC AAA-3' (sense), 5'-GAG AGA GGG AAG CTC GTT AGT GCC CCG GTA CCT CGT CC-3' (antisense).

### Transfection

MCF-7 and MDA-MB-231 cells were transfected with pCMV-*MALAT1* or pEYFP-N1 empty vector using jetPRIME transfection reagent (Polyplus-transfection, SA) according to the manufacturer’s instructions. Four hours later, the transfection medium was replaced with fresh medium containing serum. After 24 h, cells were checked for RNA expression by qPCR.

### Lentivirus Production and Infection

The lentiviral vectors were co-transfected with packaging plasmids psPAX2 and pMD2G (Addgene) into HEK293T cells. Infectious lentivirus was harvested at 24, 36, and 48 h after transfection, and filtered through 0.45 μm PVDF filters. MDA-MB-231 cells were infected with concentrated virus, and the culture medium was replaced after 24 h of incubation. Then, cells were selected by treating with puromycin for 2 days. The expression levels of *MALAT1* in cells were validated by qPCR.

### Site-Directed Mutagenesis

One of the HIF core binding motifs (hypoxia response element, HRE), located at -235 to -231 bp relative to the transcription start site of *MALAT1*, was identified. The seed region of *miR-3064-5p* was located at 1,295 ~ 1,301 and 7,853 ~ 7,859 bp of *MALAT1*. The mutations of the HRE in the pGL3-*MALAT1* promoter plasmid and the *miR-3064-5p* binding site mutations of pmiR-GLO-*MALAT1* were introduced by Biomedical Resource Core of the 1^st^ Core Facility Lab (NTU). In addition, the mutated sequences were validated by sequencing.

### Luciferase Reporter Assay

To determine the effects of HIF-1α and HIF-2α on the *MALAT1* promoter construct, HEK293T cells were seeded in 24-well plates at a density of 5×10^4^ cells/well. After 24 h, cells were transfected with 100 ng wild-type or mutant HRE firefly luciferase reporter construct, and 2 ng *Renilla* luciferase plasmid (pGL3 [hRluc/TK], kindly provided by Dr. Meng-Chun Hu, NTU) using jetPRIME (Polyplus-transfection) reagent. Also, cells were transfected with 50 ng of pcDNA3-HIF1α-P402A/P564A or 100 ng of pcDNA3-HIF2α-P405A/P531ApcDNA3. After 24 h, cells were lysed in cell lysis buffer (92.8 mM K_2_HPO_4_, 9.2 mM KH_2_PO_4_ and 0.2% Triton X-100 in ddH_2_O), and the luciferase activity was measured using the Dual-Glo luciferase reporter assay system (Promega, Fitchburg, WI, USA) and normalized to *Renilla* luciferase activity.

To determine the effect of *miR-3064-5p* on the *MALAT1* reporter construct, HEK293 cells were co-transfected with 0.025 nmol of *miR-3064-5p* mimic and 100 ng of the reporter vector containing the wild-type *MALAT1* S1 or S2 or the mutant *MALAT1* S1 or S2. After 48 h, the cells were collected, and the luciferase activities were measured using the Dual-Glo luciferase reporter assay system (Promega).

### RNA Extraction, Reverse Transcription, and Quantitative RT-PCR

Total RNA was isolated using NucleoZOL reagent (Machery-Nagel, Düren, Germany) according to the manufacturer’s instructions. One μg of total RNA was reverse-transcribed using the High-Capacity cDNA Reverse Transcription Kit (Applied Biosystems, Carlsbad, CA, USA). For reverse transcription of miRNA, SuperScript IV Reverse Transcriptase (Invitrogen, Carlsbad, CA, USA) was used with the primers listed in **Table 1**. Per the manufacturer’s instructions, 2.5% of each reaction was used as the template for qPCR with 5× HOT FIREPol^®^ EvaGreen^®^ qPCR Mix Plus (OmicsBio, New Taipei City, Taiwan), and the reactions were performed on a StepOnePlus Real-Time PCR System (Thermo Fisher, Waltham, MA, USA). The primer pairs used for detection of cDNAs are listed in **Table 1**. At last, the relative gene expression levels were normalized to 18S rRNA or *U6* using the 2^-ΔΔCt^ method.

**Table 1:** The primers for reverse transcription and quantitative RT-PCR.

Gene/miRNA	Primer	Sequence (5' to 3')
Reverse Transcription		
* miR-378c*	GTTGGCTCTGGTGCAGGGTCCGAGGTATTCGCACCAGAGCCAACCCACTC	
* miR-3064-5p*	GTTGGCTCTGGTGCAGGGTCCGAGGTATTCGCACCAGAGCCAACTTGCAC	
* miR-3150a-3p*	GTTGGCTCTGGTGCAGGGTCCGAGGTATTCGCACCAGAGCCAACCCAACC	
* miR-4325*	GTTGGCTCTGGTGCAGGGTCCGAGGTATTCGCACCAGAGCCAACTCACTG	
* miR-7855-5p*	GTTGGCTCTGGTGCAGGGTCCGAGGTATTCGCACCAGAGCCAACCCGAGC	
* U6* snRNA	CGCTTCACGAATTTGCGTGTCAT	
Quantitative RT-PCR		
* MALAT1*	Forward	GACGGAGGTTGAGATGAAGC
	Reverse	ATTCGGGGCTCTGTAGTCCT
* BCAR4*	Forward	GTTCCGATGCTTGTCTTGCTC
	Reverse	CCAAAGACGAAGATGCCAGG
* U6* snRNA	Forward	GCTTCGGCAGCACATATACTAAAAT
	Reverse	CGCTTCACGAATTTGCGTGTCAT
* GAPDH*	Forward	AACGGGAAGCTTGTCATCAATGGAAA
	Reverse	GCATCAGCAGAGGGGGCAGAG
18s rRNA	Forward	TCAACTTTCGATGGTAGTCGCCGT
	Reverse	TCCTTGGATGTGGTAGCCGTTTCT
* miR-378c*	Forward	GCGGCGGACTGGACTTGGAGTCAGAA
	Reverse	GTGCAGGGTCCGAGGT
* miR-3064-5p*	Forward	GCGGCGGTCTGGCTGTTGTGGT
	Reverse	GTGCAGGGTCCGAGGT
* miR-3150a-3p*	Forward	GCGGCGGCTGGGGAGATCCTCGA
	Reverse	GTGCAGGGTCCGAGGT
* miR-4325*	Forward	GCGGCGGTTGCACTTGTCT
	Reverse	GTGCAGGGTCCGAGGT
* miR-7855-5p*	Forward	GCGGCGGTTGGTGAGGACCCCAA
	Reverse	GTGCAGGGTCCGAGGT

### Nuclear-Cytoplasmic RNA Fractionation

To determine the subcellular localization of RNA, fractionation of nuclear and cytoplasmic RNA was performed using the Cytoplasmic & Nuclear RNA Purification Kit (Norgen Biotek, Thorold, ON, Canada). Cells were first lysed with Lysis Buffer J (Norgen Biotek), and the lysate was separated by centrifugation, after which the supernatant contained the cytoplasmic RNA and the pellet contained the nuclear RNA. Buffer SK (Norgen Biotek) and ethanol were then added to the cytoplasmic and nuclear fractions, and the solution was loaded onto a spin-column to collect RNA. The bound RNA was then washed with Wash Solution A (Norgen Biotek), and the purified RNA was eluted with Elution Buffer E (Norgen Biotek). The isolated RNA was subsequently reverse-transcribed, and the relative expression level was measured by qPCR. The pairs of primers used are listed in **Table 1**.

### RNA Fluorescence *In Situ* Hybridization

For FISH, the *MALAT1* hybridization protocol was followed from a previous publication (31). Briefly, cells were seeded onto an autoclaved glass chamber slide at a density of 3 × 10^5^ cells/well and incubated overnight. Cells were fixed by fixation buffer and incubated for 10 min at room temperature. For permeabilizing the cells, each well was soaked in 70% ethanol at 4°C overnight. Cells were then hybridized by fluorescein probes (tgaaccaaagctgcactgtg; Protech, Taiwan) labeled at the 5' end with a final concentration of 4 μM in hybridization buffer, and incubated at 37°C overnight. The next day, the hybridization buffer was removed, and cells were washed in phosphate-buffered saline (PBS) 3 times. Finally, the slide was mounted on a DAPI Fluoromount-G (SouthernBiotech). Images were acquired using a Zeiss LSM880 confocal microscope (Carl Zeiss AG, Gina, Germany).

### Colony Formation

MCF-7 cells were seeded in a six-well plate at a density of 800 cells/well. MDA-MB-231 cells were seeded in a six-well plate at a density of 500 cells/well. After 2 weeks of incubation, cells were fixed with 600 μl 75% methanol/25% acetate (Sigma) for 10 min and washed by PBS followed by staining with 0.1% crystal violet for another 10 min. Colonies with cell numbers greater than 50 were quantified using ImageJ 1.8.0 (National Institutes of Health).

### Wound Healing Assay

Cells were seeded on an Ibidi Culture-Insert (Ibidi, Martinsried, Germany) at a density of 2.5 × 10^4^ cells/reservoir and incubated overnight. The inserts were carefully removed with sterile tweezers to create a cell-free gap. The ability of cells to migrate into the gap was captured by microphotography at indicated time points and quantified using ImageJ 1.8.0 (National Institutes of Health).

### Cell Proliferation (MTT) Assay

Cells were seeded at a density of 5 × 10^4^ cells/100 μl in a 96-well plate. After seeding 12 h, 3-(4,5-Dimethylthiazol-2-yl)-2,5-diphenyltetrazolium bromide (MTT) assay solution (Sigma, St. Louis, MO, USA) (1 ml/well of a 5 mg/ml solution in PBS) was added to each well and incubated for 1 h. After incubating for 1 h, medium with MTT solution was removed and 100 μl dimethyl sulfoxide (Sigma) was added to each well to dissolve the converted purple formazan. The absorbance of formazan was measured at 570 nm using an enzyme-linked immunosorbent assay (ELISA) reader (Bio Tek, Winooski, VT, USA).

### Cell Cycle

Cells were harvested by trypsinization and fixed with cold 75% ethanol at -20°C overnight. Cells were washed with PBS and resuspended in propidium iodide (PI) (Life Technologies, Carlsbad, CA, USA) solution (20 μg PI/ml, 100% Triton-X 1μl/ml, 20ng RNase/ml in PBS) for 10 min on ice. The suspension was analyzed with a Beckman Coulter FC500 cytometer (Beckman, Brea, CA, USA).

### RNA Immunoprecipitation

To validate the interaction between RNA and RNA binding proteins, the Magna RIP Kit (Millipore) was used. Before lysis, cells were washed with cold PBS, and samples were harvested with cell scrapers. Then, cells were lysed in RIP Lysis Buffer (Millipore) with RNase inhibitor and protease inhibitor cocktail (Millipore), and the magnetic beads for immunoprecipitation were prepared according to the manufacturer’s instructions. The RNA binding protein-RNA complexes were immunoprecipitated with premade magnetic beads at 4°C with overnight agitation. After washing the beads with ice-cold RIP Wash Buffer (Millipore), the RNA binding proteins were digested with proteinase K at 55°C for 30 min with shaking. The purified RNA was isolated with TRIzol reagent (Ambion, Thermo Fisher) and reverse-transcribed, and the relative gene expression level was measured by qPCR. The pairs of primers used are listed in **Table 1**.

### Statistical Analysis

Statistical analysis was carried out using Microsoft Excel to assess differences between experimental groups. All results were reported as means ± SDs for at least 3 independent experiments. Statistical significance was analyzed by Student’s t test and expressed as a *P* value. *P* values lower than 0.05 were considered to indicate statistical significance.

## Results

**Figure 1:**
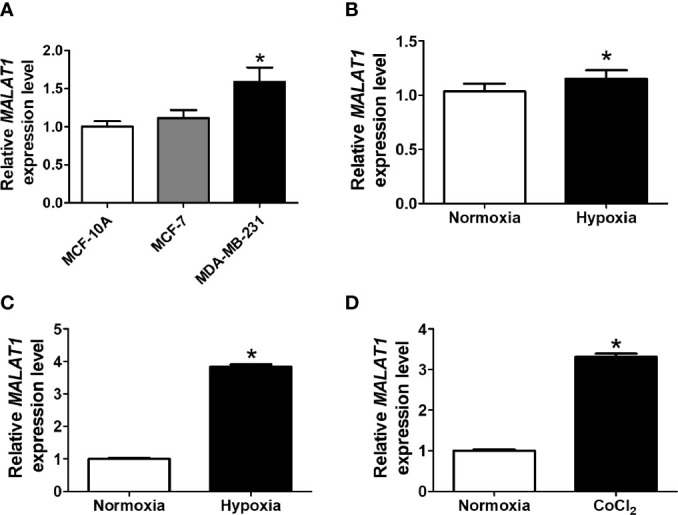
*MALAT1* is up-regulated under hypoxia in breast cancer cells. **(A)** Relative endogenous expression levels of *MALAT1* in MCF-10A, MCF-7, and MDA-MB-231 cells. The expression levels were measured by qPCR. **(B, C)** Relative expression levels of *MALAT1* in MDA-MB-231 **(B)** and MCF-7 **(C)** cells under hypoxia. **(D)** Relative expression levels of *MALAT1* in MCF-7 cells treated with CoCl_2_. Data shown are the means ± SDs (n=3). *P < 0.05.

To investigate endogenous expression levels of *MALAT1*, we first determined the expression of *MALAT1* in several breast cell lines, including MCF-10A (non-cancerous mammary gland epithelial cell), MCF-7 (luminal A cancer), and MDA-MB-231 (triple negative cancer) (**Figure 1A**). The expression levels of *MALAT1* in MDA-MB-231 cells were significantly (*P* < 0.05) higher than those in MCF-10A and MCF-7 cells. Also, the relative expression levels of *MALAT1* in MCF-7 cells under hypoxia or CoCl_2_ treatment were examined by qPCR. The relative expression of *MALAT1* in MDA-MB-231 cells (**Figure 1B**) and MCF-7 cells (**Figure 1C**) under hypoxia were significantly (*P* < 0.05) up-regulated. *MALAT1* was similarly up-regulated in MCF-7 cells treated with CoCl_2_, which mimics hypoxia (**Figure 1D**).

**Figure 2:**
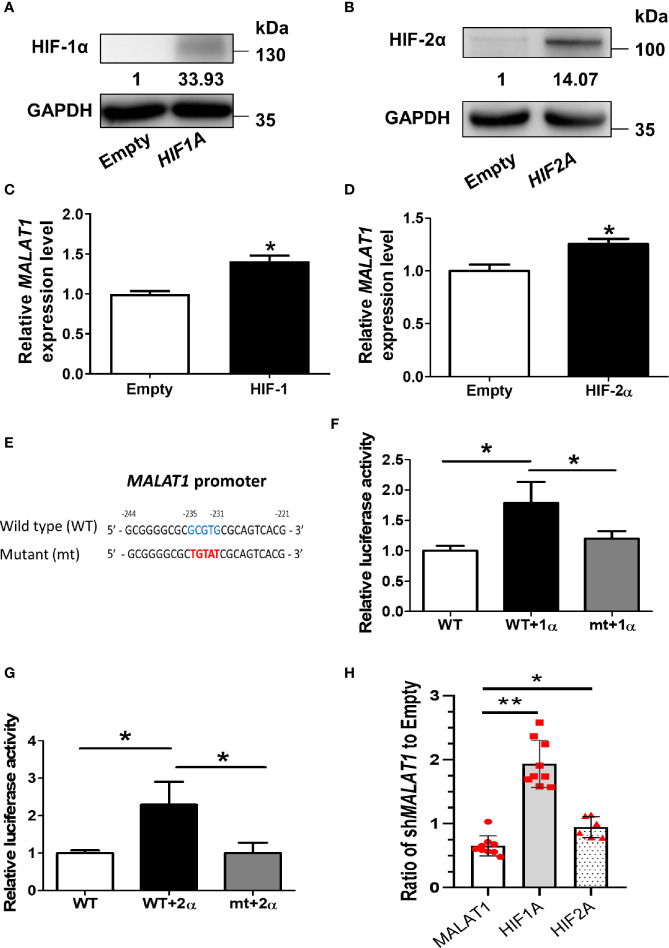
HIF-1α and HIF-2α up-regulate the transcriptional levels of *MALAT1*. **(A)** Western blot analysis of HIF-1α in MCF-7 cells over-expressing HIF-1α-P402A/P564A under normoxia. **(B)** Western blot analysis of HIF-2α in MCF-7 cells over-expressing HIF-2α-P405A/P531A under normoxia. **(C, D)** Relative expression levels of *MALAT1* in MCF-7 cells overexpressing HIF-1α-P402A/P564A **(C)** or HIF-2α-P405A/P531A **(D)**. The expression levels were measured by qPCR. **(E)** Schematic diagram of the putative HRE ([A/G]CGTG; -235 ~ -231 bp) in the promoter region (-2,000 ~ -1 bp) of *MALAT1*. **(F, G)** Luciferase reporter assays of wild-type (WT) and mutant (mt) *MALAT1* promoters in HEK-293T cells overexpressing HIF-1α-P402A/P564A **(F)** or HIF-2α-P405A/P531A **(G)**. HEK-293T cells were transfected with HIF-1α or HIF-2α expressing plasmids, firefly luciferase plasmids, and *Renilla* luciferase vectors. The relative firefly luciferase activity was measured and normalized to *Renilla* luciferase activity. All data shown are the means ± SDs (n = 3). *P < 0.05. **(H)** Relative expression levels of *MALAT1*, *HIF1A*, and *HIF2A* in MCF-7 cells transduced with sh*MALAT1*.The expression levels were measured by qPCR. Loading control: 18S rRNA. **P < 0.01. *P < 0.05.

Since *MALAT1* was significantly up-regulated under hypoxia, we hypothesized that this effect was triggered by HIFs. In order to mimic *HIF1A* or *HIF2A* overexpression, we transfected a degradation-resistant *HIF1A* mutant (pcDNA3-HIF-1α-P402A/P564A) (**Figure 2A**) or *HIF2A* mutant (pcDNA3-HIF-2α-P405A/P531A) (**Figure 2B**) into MCF-7 cells under normoxia to observe the effects of HIF-1α and HIF-2α on *MALAT1* expression. The RNA expression levels of *MALAT1* were increased significantly (*P* < 0.05) when either *HIF1A* or *HIF2A* was overexpressed (**Figures 2C, D**). To further investigate how *HIF-1A* or *HIF2A* increased *MALAT1* expression, the promoter sequence analysis revealed that there was one putative HRE ([A/G]CGTG) located at -235 to -231 bp relative to the transcription start site of *MALAT1*. Therefore, the promoter region of *MALAT1* (-2,000 to -1 bp) was inserted into the pGL3-basic vector carrying the firefly luciferase gene. In addition, to validate the putative HRE site, the HRE sequences were mutated from GCGTG to TGTAT (**Figure 2E**). Overexpression of *HIF-1A* (**Figure 2F**) or *HIF-2A* (**Figure 2G**) both increased the luciferase activity, and the HRE site mutation significantly (*P* < 0.05) decreased both luciferase activities (**Figures 2F, G**). These results indicate that both HIF-1α and HIF-2α up-regulate the transcriptional levels of *MALAT1* by binding to the HRE in its promoter. Furthermore, when *MALAT1* was knocked down by shRNA, the expression levels of *HIF-1A* and *HIF-2A* were significantly up-regulated, suggesting the negative feedback of *MALAT1* on *HIF-1A* and *HIF-2A* (**Figure 2H**).

**Figure 3:**
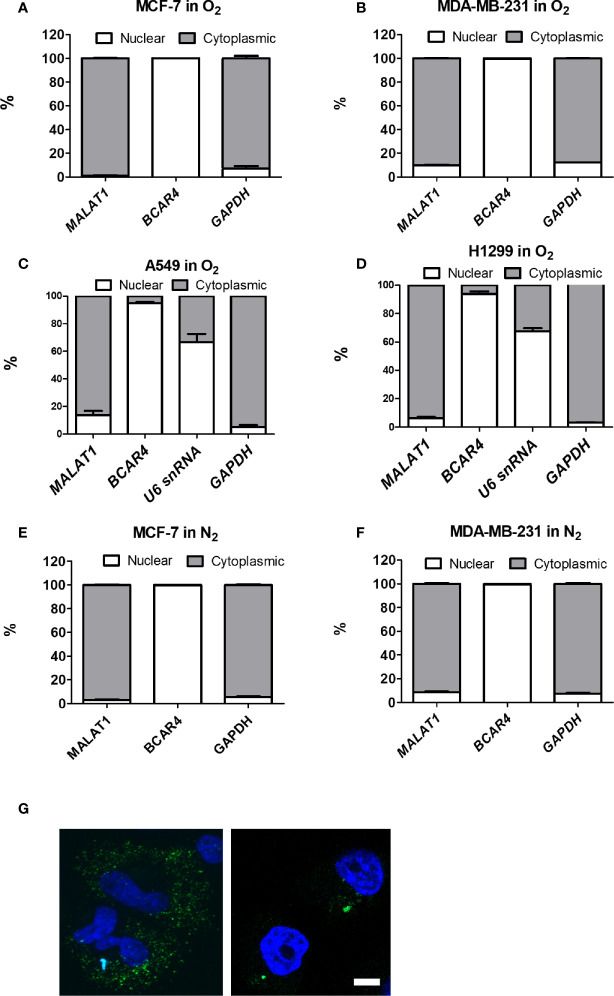
*MALAT1* is located primarily in the cytoplasm of MCF-7 and MDA-MB-231 cells under normoxia or hypoxia. **(A–D)** Cytoplasmic and nuclear distribution of *MALAT1* in breast cancer cells [MCF-7 **(A)**, MDA-MB-231 **(B)**] and lung cancer cells [A549 **(C)**, H1299 **(D)**] cells under normoxia. **(E, F)** Cytoplasmic and nuclear distribution of *MALAT1* in MCF-7 **(E)** and MDA-MB-231 **(F)** cells under hypoxia. Relative abundance of RNA was normalized to the total amount of RNA and detected by qPCR. *BCAR4* and *U6* snRNA: nuclear marker. *GAPDH*: cytoplasmic marker. Data shown are the means ± SDs (n=3). **(G)** RNA FISH of *MALAT1*. Cell nuclei were stained by Hoechst staining (blue). *MALAT1* was hybridized with *MALAT1*-FITC probes (green) in breast cancer cell lines and detected by a Zeiss LSM880 microscope. Magnification:1,000×; Scale bar: 5 μm.

To determine whether *MALAT1* could serve as a miRNA sponge to modulate cell functions, we first investigated the distribution of *MALAT1* in MCF-7 cells and MDA-MB-231 cells under normoxia (**Figures 3A, B**) and hypoxia (**Figures 3E, F**). The positive control for nuclear function was *BCAR4*, and for cytoplasmic function was *GAPDH*. Surprisingly, *MALAT1* was mainly distributed in the cytoplasm, not the nucleus. To confirm this phenomenon, we also investigated the distribution of *MALAT1* in lung cancer cell lines A549 and H1299 under normoxia, because *MALAT1* was reported to be a highly abundant nuclear transcript in these cells (**Figures 3C, D**) (18, 32, 33). Yet, similar results were observed in lung cancer cells. Nuclear-cytoplasmic RNA fractionation assays indicated that *MALAT1* was mainly located in the cytoplasm of MCF-7 cells and MDA-MB-231 cells either under normoxia or hypoxia. Furthermore, RNA FISH was used to determine the location of *MALAT1* (**Figure 3G**). These results also showed that *MALAT1* is mainly located in the cytoplasm in MCF-7 cells and MDA-MB-231 cells.

**Figure 4:**
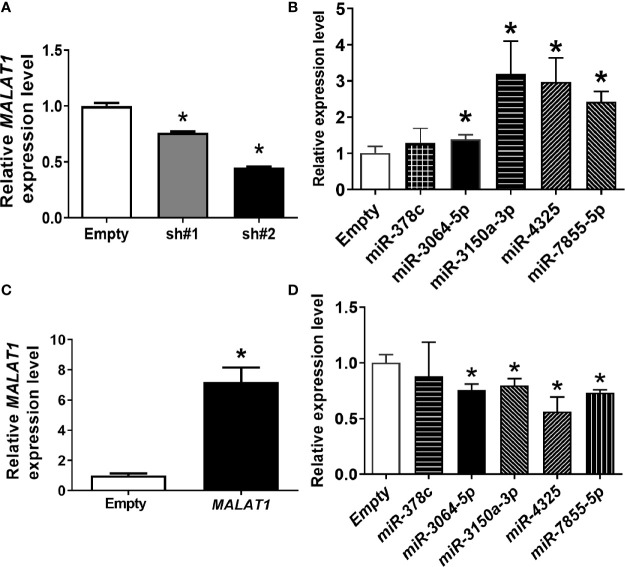
The inverse relationship between expression levels of miRNAs and *MALAT1*. **(A)** Relative expression levels of *MALAT1* in MDA-MB-231 cells were reduced with shRNA against *MALAT1* under hypoxia. The expression levels were measured by qPCR. Loading control: 18S rRNA. **(B)** Relative expression levels of miRNAs in MDA-MB-231 cells transfected with shRNA against *MALAT1*. **(C)** Relative expression levels of *MALAT1* in MCF-7 cells overexpressing *MALAT1*. Cells were transfected with pCMV-*MALAT1* and the expression levels were detected by qPCR. **(D)** Relative expression levels of miRNAs in MCF-7 cells transfected with pCMV-*MALAT1*. Data shown are the means ± SDs (n=3). *P < 0.05.

In our previous research, five differentially expressed miRNAs, including *miR-378c*, *miR-3150a-3p*, *miR-3064-5p*, *miR-4325*, and *miR-7855-5p*, were significantly up-regulated using NGS when *MALAT1* was knocked down under hypoxia (data not shown). We hypothesized that *MALAT1* may serve as a sponge to these miRNAs. To test our hypothesis, *MALAT1* was first silenced in MDA-MB-231 cells by two shRNAs targeted to different sites of *MALAT1*. *MALAT1* expression levels were significantly (*P* < 0.05) down-regulated when *MALAT1* was knocked down under hypoxia (**Figure 4A**). Next, the expression levels of these five miRNAs were validated by qPCR when *MALAT1* was knocked down under hypoxia (**Figure 4B**). The results showed that the expression levels of *miR-3064-5p*, *miR-3150*, *miR-4325*, and *miR-7855-5p* were significantly (*P* < 0.05) up-regulated when *MALAT1* was knocked down under hypoxia. Furthermore, the expression levels of *miR-3064-5p*, *miR-3150*, *miR-4325*, and *miR-7855-5p* were significantly down-regulated when *MALAT1* was overexpressed (**Figures 4C, D**). These results indicate that the expression levels of *MALAT1* are negatively correlated with those of *miR-3064-5p*, *miR-3150*, *miR-4325*, and *miR-7855-5p.*

**Figure 5:**
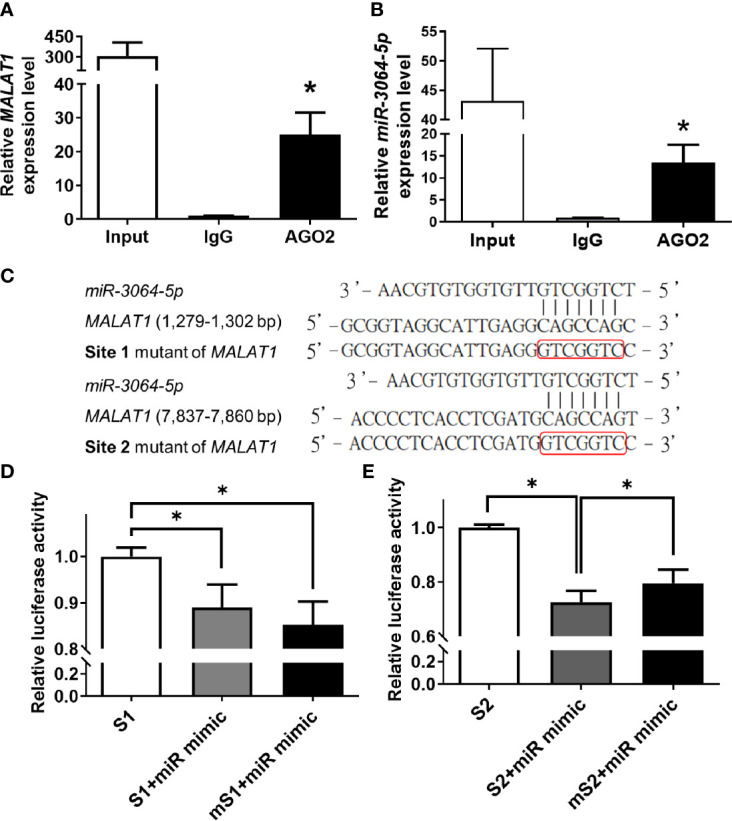
*MALAT1* binds to *miR-3064-5p* directly. **(A, B)** RIP using antibody against AGO2. The relative RNA levels of *MALAT1* **(A)** and *miR-3064-5p* **(B)** were quantified and normalized to the IgG group using qPCR. Input: positive control; IgG: negative control. **(C)** Schematic representation of firefly reporter constructs containing the fragments of Site 1 (1,279~1,302 bp) and Site 2 (7,837~7,860 bp) of *MALAT1*, and mutants with mutation at the binding sites of *miR-3064-5p*. **(D, E)** Luciferase reporter assays of *MALAT1* fragment with wild-type or mutant (m) Site 1 **(D)** or Site 2 **(E)** in cells overexpressing *miR-3064-5p* mimic. HEK-293T cells were transfected with *miR-3064-5p* mimic and firefly/*Renilla* plasmids. The relative firefly luciferase activity was measured and normalized to *Renilla* luciferase activity. Data shown are the means ± SDs (n=3). *P < 0.05.

To validate that *MALAT1* physically bound to miRNAs and served as a miRNA sponge in breast cancer, *miR-3064-5p* was chosen for further experiments, because *miR-3064-5p* has been reported to inhibit cell proliferation and invasion in ovarian cancer and to suppress angiogenesis in hepatocellular carcinoma (34, 35). First, since argonaute2 (AGO2) protein is an essential component of the miRNA-induced silencing complex (miRISC), RIP assays using anti-AGO2 antibody were performed. As shown in **Figures 5A, B**, *MALAT1* and *miR-3064-5p* were both significantly (*P* < 0.05) enriched with AGO2 immunoprecipitation compared with IgG control group. Next, sequence analysis of *MALAT1* revealed two putative binding sites for *miR-3064-5p*, located at 1,279~1,302 bp (Site 1) and 7,837~7,860 bp (Site 2) relative to the transcription start site. Therefore, wild-type or mutant versions of the regions of *MALAT1* (1,048~1,547 or 7,607~8,106 bp) containing the putative binding sites of *miR-3064-5p* were inserted into the pmiR-GLO vector (**Figure 5C**), and luciferase reporter assays were performed. Overexpression of *miR-3064-5p* mimic decreased the *MALAT1* promoter-mediated luciferase activity, but the luciferase activity was rescued only by the site 2 mutation, not the site 1 mutation (**Figures 5D, E**). Taken together, these results indicate that *miR-3064-5p* regulates *MALAT1* directly by binding at 7,837~7,860 bp of *MALAT1*.

**Figure 6:**
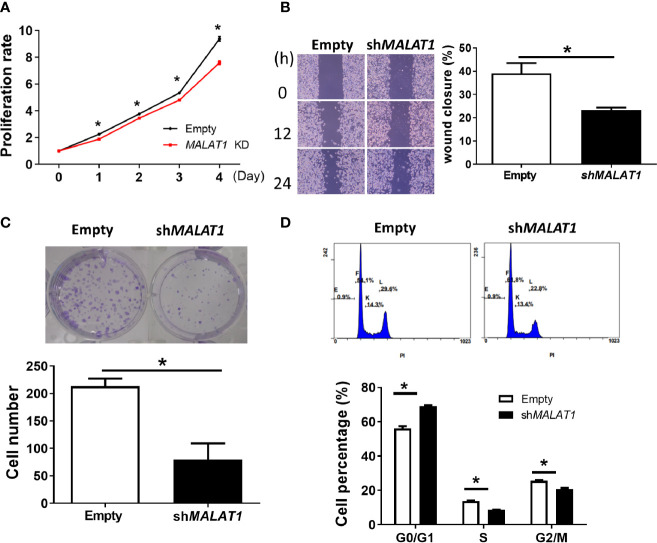
Knockdown of *MALAT1* decreases proliferation and metastasis in MDA-MB-231 cells. **(A)** Measurement of cell proliferation using MTT assays. Cell growth was measured in MDA-MB-231 cells transduced with lentivirus which expresses shRNA against *MALAT1*. The proliferation rate was normalized to day zero. **(B)** Wound healing assay. Left: Representative pictures of wound healing assays. After 24 h of transduction, wound healing was photographed at 0, 12, and 24 h. Right: Migration ability was quantified as reduction in wound size at 24 h. **(C)** Colony formation assay. Top: Representative pictures of colony formation assays. Colonies with cell numbers more than fifty were counted. Bottom: Quantification of results. **(D)** Cell cycle distribution by flow cytometry. Top: Representative diagrams of flow cytometry. After 48 h of transfection, cells were harvested and stained with PI. Bottom: Quantification of results as the percentage of cells in each phase of the cell cycle. All data shown are the means ± SDs (n = 3). *P < 0.05.

Since more expression of *MALAT1* was observed in MDA-MB-231 cells, we studied the functional effects of *MALAT1* knockdown on cell proliferation, cell migration, colony formation, and cell cycle distribution in MDA-MB-231 cells. The effect of *MALAT1* on cell proliferation was examined by MTT assays. MDA-MB-231 cells with *MALAT1* knockdown had significantly (*P* < 0.05) decreased cell proliferation (**Figure 6A**). Next, the effects of *MALAT1* on cell migration were examined by wound healing assays. MDA-MB-231 cells with *MALAT1* knockdown had significantly (*P* < 0.05) decreased cell migration (**Figure 6B**). Long term effects of *MALAT1* on cell proliferation were observed through colony formation assays. MDA-MB-231 cells with *MALAT1* knockdown had significantly decreased colony numbers (**Figure 6C**). Lastly, the effects of *MALAT1* on cell cycle distribution were examined by flow cytometry. In *MALAT1* knockdown cells, the percentage of cells in G1 phase significantly increased as compared to the empty vector controls (**Figure 6D**). Conversely, the percentage of cells in the S and G2/M phases decreased. Taken together, these data show that MDA-MB-231 cells with *MALAT1* knockdown had significantly decreased ability to multiply, migrate, and colonize, suggesting that breast tumor malignancy could be mediated by *MALAT1*.

**Figure 7:**
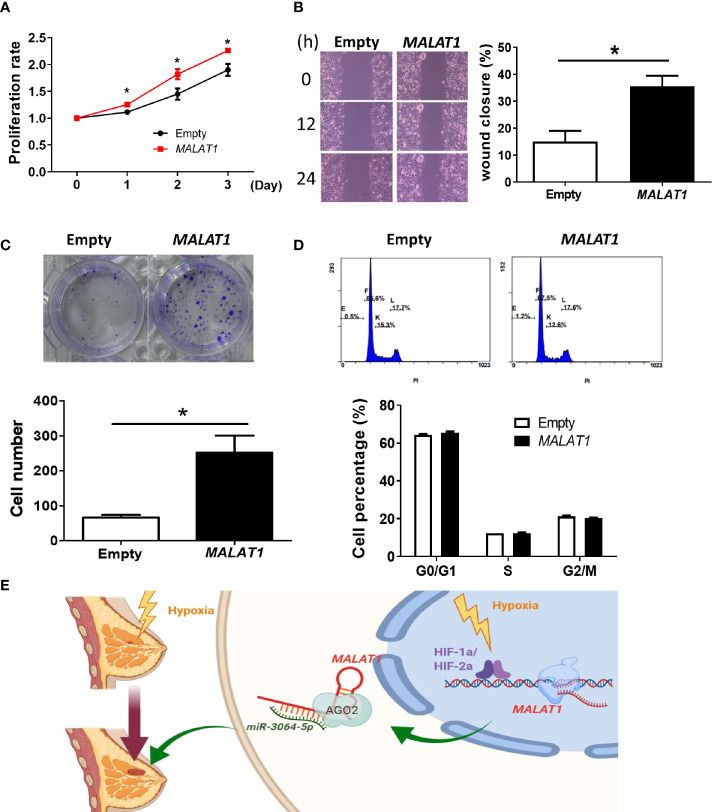
Overexpression of *MALAT1* promotes proliferation and migration in MCF-7 cells. **(A)** Measurement of cell proliferation using MTT assays. Cell growth was measured in MCF-7 cells overexpressing *MALAT1*. The proliferation rate was normalized to day zero. **(B)** Wound healing assay. Left: Representative pictures of wound healing assays. After 24 h of transfection, wound healing was photographed at 0, 12, and 24 h. Right: Migration ability was quantified as reduction in wound size at 24 h. **(C)** Colony formation assay. Top: Representative pictures of colony formation assays. Colonies with cell numbers more than fifty were counted. Bottom: Quantification of results. **(D)** Cell cycle distribution by flow cytometry. Top: Representative diagrams of flow cytometry. After 48 h of transfection, cells were harvested and stained with PI. Bottom: Quantification of results as the percentage of cells in each phase of the cell cycle. **(E)** A proposed model for illustrating the regulatory mechanisms and function of hypoxia-induced lncRNA *MALAT1* in breast cancer cells. All data shown are the means ± SDs (n = 3). *P < 0.05.

Finally, considering that the endogenous expression levels of *MALAT1* in MCF-7 cells were lower than in MDA-MB-231 cells, we also studied the functional effects of overexpressing *MALAT1* in MCF-7 cells. MCF-7 cells with *MALAT1* overexpression had significantly increased cell proliferation (**Figure 7A**), cell migration (**Figure 7B**), and ability to form colonies (**Figure 7C**). However, in *MALAT1* overexpressing cells, the distribution of cells in each phase of the cell cycle showed no significant differences as compared to empty controls (**Figure 7D**). These data confirm that *MALAT1* overexpression leads to characteristics associated increased tumor malignancy in MCF-7 cells.

Taken together, these data suggested that hypoxia-responsive long non-coding *MALAT1* could be transcriptionally activated by HIF-1α and HIF-2α, act as a miRNA sponge of *miR-3064-5p*, and promote tumor growth and migration in breast cancer cells (**Figure 7E**).

## Discussion

In this study, we demonstrated that *MALAT1* was up-regulated under hypoxia in breast cancer cells. Luciferase reporter assays showed that *HIF-1A* and *HIF-2A* both increased the transcriptional activity of *MALAT1*. Next, the nuclear and cytoplasmic fractionation assays and FISH indicated that *MALAT1* was mainly located in the cytoplasm. Four hypoxia-responsive miRNAs, including *miR-3064-5p*, *miR-3150*, *miR-4325*, and *miR-7855-5p*, had reverse relationships with the expression of *MALAT1*. In addition, RIP assays using antibody against AGO2 showed that *MALAT1* served as a miRNA sponge for *miR-3064-5p*. Lastly, functional assays revealed that *MALAT1* could promote breast cancer cell aggressiveness, by increasing proliferation and migration and altering cell cycle distribution.

LncRNAs are known to play a crucial role in carcinogenesis (36). For example, the lncRNA *PRLB* promotes tumorigenesis through regulating the *miR-4766-5p/SIRT1* axis (37). LncRNA *HIFCAR/MIR31HG* was found to be a HIF-1α co-activator that promoted oral cancer progression (38). LncRNA *UCA1* promoted proliferation, migration, and immune escape and inhibited cell apoptosis in gastric cancer (39). Our results revealed that the endogenous expression levels of *MALAT1* in MDA-MB-231 metastatic breast cancer cells were higher than in normal MCF-10A breast epithelial cells and in less aggressive MCF-7 breast cancer cells (**Figure 1A**), suggesting that *MALAT1* plays a role in the oncogenic characteristics in breast cancer. Another study reported similar results, that *MALAT1* expression levels were significantly higher in tumor tissues as compared with adjacent noncancerous tissues (40).

So far, some lncRNAs have been confirmed to respond to hypoxia in several malignant tumors and to regulate gene expression to adjust to microenvironments deficient in oxygen (41–44). Recent RNA-seq results showed that >100 lncRNAs, including *H19*, *MIR210HG*, and *MALAT1*, were up-regulated in human umbilical vein endothelial cells under hypoxia (45). We found that *MALAT1* was also up-regulated in hypoxia in breast cancer cells (**Figure 1**). Similarly, *MALAT1* was reported to be induced in hypoxia and to regulate polypyrimidine tract-binding protein (PTB)-associated splicing factor transcriptionally in A549 lung cancer cells (46). In hypoxia, HIF-1 is known to function as an oxygen-regulated transcriptional activator that is expressed ubiquitously and plays essential roles in mammalian development, physiology, and disease pathogenesis (47–49). Unlike the ubiquitously expressed HIF-1α, HIF-2α is mainly expressed in endothelial cells (50). Some genes are regulated only by HIF-2α and not HIF-1α in cancer. In our results, ectopic expression of both HIF-1α or HIF-2α increased *MALAT1* expression (**Figures 2C, D**). Furthermore, promoter analysis revealed that there is one putative HRE in the *MALAT1* promoter (**Figure 2E**). Our results revealed that both HIF-1α and HIF-2α could up-regulate expression of *MALAT1* by binding to its promoter. Several studies have revealed evidence that *MALAT1* promotes arsenite-induced glycolysis in human hepatic L-02 cells through HIF-1α stabilization (51). Our results suggest that HIF-2α could also regulate cell functions by modulating the expression of noncoding RNAs, such miRNA and lncRNA. This is consistent with the finding that the HIF-2α/*MALAT1*/*miR-216b* axis up-regulated autophagy to promote multi-drug resistance in hepatocellular carcinoma cells (52).

Furthermore, the negative feedback loop of *MALAT1* on *HIF-1A* and *HIF-2A* was discovered in this study (**Figure 2H**). *MALAT1* was reported to increase HIF-1α expression by blocking the ubiquitin-proteasome pathway in arsenite-induced glycolysis (53). Also, a positive feedback loop between *MALAT1* and *HIF-2α* was discovered in arsenite induced hepatocellular carcinomas (54). Our results extend the current understanding regarding the reciprocal regulation between *MALAT1* and *HIF-1A* as well as *MALAT1* and *HIF-2A*.

Initially, *MALAT1* was identified as being up-regulated in primary human non-small cell lung cancer cells with heightened metastatic potential (15). Also early in its history, *MALAT1* was found to be abundant in neurons and to modulate synaptogenesis by regulating gene expression in cultured hippocampal neurons (55). In one study, *MALAT1* was called noncoding nuclear-enriched abundant transcript 2 (*NEAT2*), indicating its nuclear abundance in several cancer cell lines, and its role in alternative splicing regulation (46). For a long time, *MALAT1* was considered to be a nuclear marker in certain cancer cell lines (18, 32, 33), especially cancers with aggressive metastatic tumors (15), and it has been shown to be involved in proliferation and invasion of lung cancer cells (56) and cervical cancer cells (57). However, the results of our nuclear-cytoplasmic RNA fractionation assays indicated that *MALAT1* was mainly located in the cytoplasm of MCF-7, MDA-MB-231, A549, and H1299 cells, under either normoxia or hypoxia (**Figure 3**). These results are consistent with recent studies showing that *MALAT1* was located in the cytoplasm in human hepatocellular carcinoma cells, monocytes, and human pulmonary microvascular endothelial cells (29, 30, 35). Therefore, the labeling of *MALAT1* as a nuclear marker should be done with the caveat that this status is dependent on the cell type.

LncRNAs and miRNAs can work cooperatively to mediate gene expression *via* post-transcriptional mechanisms. Since *MALAT1* was mainly in the cytoplasm of breast cancer cells, we hypothesized that *MALAT1* may serve as a miRNA sponge. In this study, we showed that four hypoxia-responsive miRNAs (*miR-3064-5p*, *miR-3150a-3p*, *miR-4325*, and *miR-7855-5p*) had negative correlation with the expression *MALAT1* (**Figure 4**). Furthermore, *MALAT1* was enriched in RIP assays using antibody against AGO2 (**Figure 6A**). The results of luciferase reporter assays also indicated that *miR-3064-5p* regulated *MALAT1* directly by binding at 7,837~7,860 bp relative to the transcription start site of *MALAT1*. However, mutation of this site on *MALAT1* did not fully rescue the luciferase activity (**Figure 5E**), suggesting that *miR-3064-*5p may target other sites of *MALAT1.* Other references have also reported that *MALAT1* could serve as a miRNA sponge. For example, *MALAT1* functioned as a competing endogenous RNA (ceRNA) by sponging *miR-3064-5p*, which alleviated the suppressive effect on angiogenesis in human hepatocellular carcinoma *via* the FOXA1/CD24/Src pathway (35). *MALAT1* targeted *miR-150-5p* to exacerbate acute respiratory distress syndrome by upregulating ICAM-1 expression (29). *MALAT1* bound *miR-23a* to suppress inflammation in septic mice (30).

In our experiments, *MALAT1* was shown to promote cell proliferation and migration in MFC7 and MDA-MB-231 cells (**Figures 6**, **7**). When *MALAT1* was knocked down in MDA-MB-231 cells, the percentage of G1 phase of cell cycle significantly increased, indicating that silencing of *MALAT1* resulted in G1 arrest. However, the downstream genes of *MALAT1/miRNA-3064-5p* that influence the functions of breast cancer cells are still unknown. In the future, we can use predictive tools to investigate the target genes of *miRNA-3064-5p* and validate these genes experimentally. Also, an animal model is needed to confirm the role of the *MALAT1/miR-3064-5p* pathway in breast cancer. Since the suppressive role of *Malat1* on metastatic ability of breast cancer in mouse has been reported (58), more experiments in animal studies and clinical trials are still warranted to explore the *MALAT1* pathway as a therapeutic target for breast cancer.

## Data Availability Statement

The raw data supporting the conclusions of this article will be made available by the authors, without undue reservation.

## Ethics Statement

The study was approved by the Biosafety Committee of College of Medicine, National Taiwan University [BG1050086].

## Author Contributions

C-HS and L-CL conceived and designed the experiments. C-HS performed the experiments. C-HS, L-LC, and T-PL analyzed the data. L-HC, M-HT, and EC contributed reagents, materials, and/or analysis tools. C-HS and L-CL wrote the paper. All authors contributed to the article and approved the submitted version.

## Funding

This work was supported by a grant from the Ministry of Science and Technology [MOST 109-2320-B-002-016-MY3]. These funding sources had no role in the design of this study and will not have any role during its execution, analyses, interpretation of the data, or decision to submit results.

## Conflict of Interest

The authors declare that the research was conducted in the absence of any commercial or financial relationships that could be construed as a potential conflict of interest.
